# What are the long-term symptoms and complications of COVID-19: a protocol for a living systematic review

**DOI:** 10.12688/f1000research.27284.1

**Published:** 2020-12-14

**Authors:** Melina Michelen, Louise Sigfrid, Lakshmi Manoharan, Natalie Elkheir, Claire Hastie, Margaret O'Hara, Jake C. Suett, Vincent Cheng, Amanda Burls, Carol Foote, Charitini Stavropoulou

**Affiliations:** 1 School of Health Sciences, City, University of London, London, UK; 2 ISARIC Global Support Centre, Centre for Tropical Medicine and Global Health, University of Oxford, Oxford, UK; 3 Department of Clinical Research, Faculty of Infectious and Tropical Diseases, London School of Hygiene and Tropical Medicine, London, UK; 4 Long Covid Support, Birmingham, UK; 5 Queen Elizabeth Hospital Kings Lynn, Norfolk, UK; 6 Bristol Medical School, University of Bristol, Bristol, UK; 7 Independent Editor, Soquel, California, USA

**Keywords:** Living systematic review, COVID-19, long covid, lasting effects

## Abstract

Although the majority of patients with COVID-19 will experience mild to moderate symptoms and will recover fully, there is now increasing evidence that a significant proportion will experience persistent symptoms for weeks or months after the acute phase of the illness. These symptoms include, among others, fatigue, problems in breathing, lack of smell and taste, headaches, and also depression and anxiety. It has also become clear that the virus has lasting effects not only on the respiratory system but also on other parts of the body, including the heart, liver, and the nervous system.

In this paper we present a protocol for a living systematic review that aims to synthesize the evidence on the prevalence and duration of symptoms and clinical features of post-acute COVID-19 and its long-term complications.

The living systematic review will be updated regularly, initially monthly with update cycles under continuous review as the pace of new evidence generated develops through the pandemic. We will include studies that follow up with COVID-19 patients who have experienced persistent mild, moderate or severe symptoms, with no restrictions regarding country, setting, or language.

We will use descriptive statistics to analyse the data and our findings will be presented as infographics to facilitate transcription to lay audiences. Ultimately, we aim to support the work of policy makers, practitioners, and patients when planning rehabilitation for those recovering from COVID-19.

The protocol has been registered with PROSPERO ( CRD42020211131, 25/09/2020).

## Background

More than six months into the pandemic, our knowledge around COVID-19 continues to develop rapidly. The range of documented COVID-19 infections vary from asymptomatic to severe, but the vast majority of patients experience mild to moderate symptoms and do not require hospitalisation ^1^. We have previously conducted a rapid review of the literature to identify which symptoms and signs might differentiate mild and moderate from severe COVID-19 ^2^. Since then, and as more data are being gathered, there is increasing evidence of a “long-tail” of COVID-19 illness, but limited information about the range and duration of symptoms experienced ^3^ or longer term health complications. A community app developed at King’s College London, which tracks self-reported symptoms, has shown that about one in ten will be sick for three weeks or more ( https://covid.joinzoe.com/post/covid-long-term). Some individuals with COVID-19 have reported “fatigue, headaches and tingling nerves” that lasted months after symptom onset ^4^. A recent longitudinal cohort of 143 patients followed after hospitalisation from COVID-19 in Italy reported that 87% had at least one ongoing symptom, most (55%) reporting three or more, at 60 day follow up. Fatigue (53%), dyspnoea (43%), joint pain (27%) and chest pain (22%) were the most common ongoing symptoms ^5^, but there is a variety of other symptoms and complications that have been reported including neurocognitive difficulties, muscle pains and weakness, gastrointestinal upset, rashes, metabolic disruption, thromboembolic conditions and mental health conditions ^6^. A prolonged course of illness has also been reported among people with mild COVID-19 who did not require hospitalisation ^3, 7, 8^.

The evidence to date remains fragmented as to the onset of symptoms and clinical features, how long symptoms may last, how this relates to the severity of the initial illness, and further lasting impacts to health. A better understanding of patients’ projected recovery from COVID-19 is helpful to patients, healthcare professionals, policymakers and commissioners. The clinical management of persisting symptoms of COVID-19 has started to be addressed in the clinical literature ^6^ and NHS England has issued guidance for the multisystem needs of patients recovering from COVID-19 ^9^. Our findings could help identify people requiring additional rehabilitation services and, where necessary, specialist referral to establish a secondary cause of their symptoms. Our findings will also be relevant to organisations such as NHS England, which have recently launched an online COVID-19 rehab service supporting patients suffering long-term effects of the disease ( https://www.yourcovidrecovery.nhs.uk/) or the British Society of Immunologists, which recently released a briefing note recommending research into the long-term immunological health consequences of COVID-19 ^10^.

The aim of this review is to synthesize the evidence on the prevalence and duration of symptoms and clinical features of post-acute COVID-19 and its long-term complications. This will inform clinical and public health management, prevention and rehabilitation policies.

## Methods

To address the aim of this study we will conduct a living systematic review (LSR). LSRs are used in areas where research evidence is emerging rapidly, current evidence is uncertain, and new research may influence policy or practice decisions ^11^. These are all features of COVID-19 research, where much about the long-term effects of the disease are still unknown and policy makers are calling for more evidence. The review will be initially updated monthly, with update cycles under continuous review as the pace of new evidence generated develops through the pandemic. We aim to continue to update the review for up to two years. Our study methodology has been developed and strengthened through consultation with Long Covid Support (a patient support network). 

### Inclusion/exclusion criteria

We will include studies that meet the follow criteria:

- Studies of patients with COVID-19 who have persistent mild, moderate or severe symptoms as defined by the article authors
- Studies following up with COVID-19 patients
- Peer reviewed articles published since 1 ^st^ January 2020
- No restriction regarding country, setting or language

We will exclude:

- Studies that focus only on acute COVID-19
- Editorials and opinion papers

### Search strategy

A search of the following databases will be conducted: Pubmed and CINAHL through the EBSCO database host for general health peer-reviewed articles and Global Health for global peer-reviewed articles through the Ovid database host. In addition, we will search Cochrane for relevant systematic reviews and Google Scholar for grey literature including pre-prints. We will also look at the WHO Global Research Database on COVID-19 and LitCOVID as two databases that bring together evidence on COVID-19 from a worldwide dataset. Finally, we will contact experts in the field and use social media to identify relevant studies.

Data will be managed using the review software Rayyan ^12^.

### Key search terms

We will search using controlled subject headings and keywords of the following concepts: Terms related to 1) COVID-19 OR COVID OR SARS-CoV-2; 2) symptoms OR clinical features OR signs OR characteristics OR sequelae OR complications; 3) long-term OR post-acute OR long-tail OR persistent OR chronic COVID OR long COVID OR post discharge OR prolonged symptoms OR long haul. The search terms were piloted on Pubmed and CINAHL through the EBSCO database host the week starting 14 ^th^ September 2020 to ensure that recent high profile research articles on long covid were included. No important studies were missed.

An example is shown below:

**Table:** 

MEDLINE Search	
S1. COVID-19 OR OR covid OR SARS-CoV-2. ab	31,903
S2. symptom* OR "clinical features" OR signs OR characteristic* OR sequelae OR complication*.ab	188,243
S3. "long-term Covid" OR long-term N2 consequence* OR "long-term impact" OR "long-term effect" OR "post- acute" OR long-tail OR persist* OR "chronic-COVID" OR "long-COVID" OR post-discharge OR postdischarge OR "prolonged symptom" OR "long-haul" .ab	25,598
S4. S1 AND S2 AND S3	309

### Screening

Initial screening of titles and abstracts as well as full text screening against the inclusion criteria will be done by two reviewers. Disagreements for inclusion will be resolved by consensus. Where disagreements cannot be resolved, a third researcher will review the papers to make the final decision.

### Critical appraisal checklist

We will be using the Hoy *et al.* checklist ^13^ to critically appraise the studies included in the review.

### Data extraction

The following information will be extracted from each study based on the extraction form used for our initial review ^2^: study aim, country of study, setting, method, study design and population size and characteristics, types and frequency of symptoms reported, onset and duration of symptoms. Data extraction will be performed by one reviewer and checked by a second reviewer. Disagreements will be resolved through discussion and consensus.

### Data analysis

We will use descriptive statistics to summarise the types of symptoms, their frequency and duration. We will perform subgroup analysis on the basis of age, sex, comorbidities and severity of the disease. The data will be presented as infographics to facilitate transcription to lay audiences.

### Protocol registration

This protocol report is structured according to the Preferred Reporting Items for Systematic Reviews and Meta-Analyses Protocols (PRISMA-P) statement guidelines ^14^, was registered with PROSPERO ( CRD42020211131, 25 September 2020). The protocol will be updated as we progress with the living review as and if needed. CS is the guarantor for this study.

## Data availability

### Underlying data

No underlying data are associated with this article.

### Reporting guidelines

Figshare: PRISMA-P checklist for “What are the long-term symptoms and complications of COVID-19: a protocol for a living systematic review”. https://doi.org/10.25383/city.13187456.v1 ^15^.
