# What are the long-term symptoms and complications of COVID-19: a protocol for a living systematic review

**DOI:** 10.12688/f1000research.27284.2

**Published:** 2021-08-27

**Authors:** Melina Michelen, Vincent Cheng, Lakshmi Manoharan, Natalie Elkheir, Drew Dagens, Claire Hastie, Margaret O'Hara, Jake C. Suett, Dania Dahmash, Paulina Bugaeva, Ishmaela Rigby, Daniel Munblit, Eli Harriss, Amanda Burls, Carol Foote, Janet Scott, Gail Carson, Piero Olliaro, Louise Sigfrid, Charitini Stavropoulou

**Affiliations:** 1 School of Health Sciences, City, University of London, London, UK; 2 Bristol Medical School, University of Bristol, Bristol, UK; 3 ISARIC Global Support Centre, Centre for Tropical Medicine and Global Health, University of Oxford, Oxford, UK; 4 Department of Clinical Research, Faculty of Infectious and Tropical Diseases, London School of Hygiene and Tropical Medicine, London, UK; 5 Long Covid Support, Birmingham, UK; 6 Queen Elizabeth Hospital Kings Lynn, Norfolk, UK; 7 Department of Paediatrics and Paediatric Infectious Diseases, Institute of Child’s Health, Sechenov First Moscow State Medical University (Sechenov University), Moscow, Russian Federation; 8 Inflammation, Repair and Development Section, National Heart and Lung Institute, Faculty of Medicine, Imperial College London, London, UK; 9 Research and Clinical Center for Neuropsychiatry, Moscow, Russian Federation; 10 Bodleian Health Care Libraries, University of Oxford, Oxford, UK; 11 Independent Editor, Soquel, California, USA; 12 MRC-University of Glasgow Centre for Virus Research, Glasgow, UK

**Keywords:** Living systematic review, COVID-19, long covid, lasting effects

## Abstract

Although the majority of people with Covid-19 will experience mild to moderate symptoms and will recover fully, there is now increasing evidence that a significant proportion will experience persistent symptoms for months after the acute phase of the illness. These symptoms include, among others, fatigue, problems breathing, lack of smell and taste, headaches, and depression and anxiety. It is also clear the virus has lasting fluctuating multiorgan sequelae, including affecting not only the respiratory system but also the heart, liver, and nervous system.

We present a protocol for a living systematic review that aims to synthesize the evidence on the prevalence and characteristics of post-acute COVID-19.

The living systematic review will be updated regularly, approximately every 6 months, as new evidence emerges. We will include studies that follow up at least 100 people with Covid-19 at 12 or more weeks post Covid-19 onset, with no restrictions regarding country, setting, or language.

We will use descriptive statistics and, for outcomes reported in two or more studies, we will use meta-analyses to estimate prevalence with 95% confidence intervals (CIs) using the exact method. Heterogeneity between estimates will be assessed using the I2 statistic. Our findings will also be presented as infographics to facilitate transcription to lay audiences. Ultimately, we aim to support the work of policy makers, practitioners, and patients when planning rehabilitation for those recovering from Covid-19.

The protocol has been registered with PROSPERO ( CRD42020211131, 25/09/2020).

## Background

The range of documented Covid-19 infections vary from asymptomatic to severe, but the vast majority of patients experience mild to moderate symptoms and do not require hospitalisation ^1^. We have previously conducted a rapid review of the literature to identify which symptoms and signs might differentiate mild and moderate from severe Covid-19 ^2^. Since then, and as more data are being gathered, there is increasing evidence of a “long-tail” of Covid-19 illness, but limited information about the range and duration of symptoms experienced ^3^ or longer term health complications. A community app developed at King’s College London, which tracks self-reported symptoms, has shown that about one in ten will be sick for three weeks or more ( https://covid.joinzoe.com/post/covid-long-term). Some individuals with Covid-19 have reported “fatigue, headaches and tingling nerves” that lasted months after symptom onset ^4^. A recent longitudinal cohort of 143 patients followed after hospitalisation from Covid-19 in Italy reported that 87% had at least one ongoing symptom, most (55%) reporting three or more, at 60 day follow up. Fatigue (53%), dyspnoea (43%), joint pain (27%) and chest pain (22%) were the most common ongoing symptoms ^5^, but there is a variety of other symptoms and complications that have been reported including neurocognitive difficulties, muscle pains and weakness, gastrointestinal upset, rashes, metabolic disruption, thromboembolic conditions and mental health conditions ^6^. A prolonged course of illness has also been reported among people with mild Covid-19 who did not require hospitalisation ^3, 7, 8^.

The evidence to date remains fragmented as to the onset of symptoms and clinical features, how long symptoms may last, how this relates to the severity of the initial illness, and further lasting impacts to health. A better understanding of patients’ projected recovery from Covid-19 is helpful to patients, healthcare professionals, policymakers and commissioners. The clinical management of persisting symptoms of Covid-19 has started to be addressed in the clinical literature ^6^ and NHS England has issued guidance for the multisystem needs of patients recovering from Covid-19 ^9^. Our findings could help identify people requiring additional rehabilitation services and, where necessary, specialist referral to establish a secondary cause of their symptoms. Our findings will also be relevant to organisations such as NHS England, which have recently launched an online Covid-19 rehab service supporting patients suffering long-term effects of the disease ( https://www.yourcovidrecovery.nhs.uk/) or the British Society of Immunologists, which recently released a briefing note recommending research into the long-term immunological health consequences of Covid-19 ^10^.

The aim of this review is to synthesize and continually update the evidence on the characteristics, including prevalence and duration of symptoms and clinical features of post-acute COVID-19, as well as risk factors for developing Long Covid. This will inform clinical and public health management, prevention, and rehabilitation policies.

## Methods

To address the aim of this study we will conduct a living systematic review (LSR). LSRs are used in areas where research evidence is emerging rapidly, current evidence is uncertain, and new research may influence policy or practice decisions ^11^. These are all features of Covid-19 research, where much about the long-term effects of the disease are still unknown and policy makers are calling for more evidence. The review will be updated approximately every six months, with update cycles under continuous review as the pace of new evidence generated develops through the pandemic. We aim to continue to update the review for up to two years. Our study methodology has been developed and strengthened through consultation with Long Covid Support (a patient support network).

### Inclusion/exclusion criteria

We will include studies that meet the follow criteria:

• Studies following up with at least 100 people with suspected, laboratory confirmed, and/or clinically diagnosed Covid-19
• Studies assessing symptoms or outcomes at 12 or more weeks post Covid-19 onset
• Peer reviewed articles published since 1 January 2020
• No restriction regarding country, setting, or language

We will exclude:

• Studies that focus only on acute Covid-19
• Editorials and opinion papers

### Search strategy

A search of the following databases will be conducted: Pubmed and CINAHL through the EBSCO database host for general health peer-reviewed articles and Global Health for global peer-reviewed articles through the Ovid database host. In addition, we will search Google Scholar for grey literature. We will also conduct complementary searches using the WHO Global Research Database on Covid-19 and LitCOVID as two databases that bring together evidence on Covid-19 from a worldwide dataset. A ‘backwards’ snowball search will be conducted for the references of systematic reviews. Finally, we will contact experts in the field and use social media to identify relevant studies.

We will search using controlled subject headings and keywords of the following concepts: Terms related to 1) COVID-19 OR COVID OR SARS-CoV-2; 2) symptoms OR clinical features OR signs OR characteristics OR sequelae OR complications; 3) long-term OR post-acute OR long-tail OR persistent OR chronic COVID OR long COVID OR post discharge OR prolonged symptoms OR long haul. The search terms were piloted on Pubmed and CINAHL through the EBSCO database host the week starting 14 ^th^ September 2020 to ensure that high profile research articles on long covid were included. No important studies were missed.

An example is shown below:

**Table:** 

MEDLINE Search (16/3/2021)	
S1. COVID-19 OR OR covid OR SARS-CoV-2. ab	
S2. symptom* OR "clinical features" OR signs OR characteristic* OR sequelae OR complication*.ab	
S3. "long-term Covid" OR long-term N2 consequence* OR "long-term impact" OR "long-term effect" OR "post- acute" OR long-tail OR persist* OR "chronic-COVID" OR "long-COVID" OR post-discharge OR postdischarge OR "prolonged symptom" OR "long-haul" .ab	
S4. S1 AND S2 AND S3	1952

### Screening

Search results will be managed and screened using a review online platform, Rayyan ^12^. Initial screening of titles and abstracts as well as full text screening against the inclusion criteria will be done by two reviewers independently. Non-English articles will be translated using Google Translate or reviewed by a reviewer with good knowledge of the language. Disagreements for inclusion will be resolved by consensus. Where disagreements cannot be resolved, a third researcher will review the papers to make the final decision.

### Risk of bias

We will be using the Hoy *et al.* checklist ^13^ to critically appraise the studies included in the review, a validated tool for assessing risk of bias in prevalence studies.

### Data extraction

The following information will be extracted from each study based on an extraction form informed by a previous review ^2^: study aim, country of study, setting, method, study design, population size and characteristics, types and frequency of symptoms reported, onset and duration of symptoms, treatment and possible risk factors. Data extraction will be performed by one reviewer and checked by a second reviewer. Disagreements will be resolved through discussion and consensus.

### Outcomes

The primary outcome is to characterise the prevalence of symptoms and complications of long term Covid-19 in different populations. Secondary outcomes include diagnostics and risk factors for developing different sequelae.

### Data analysis

We will use descriptive analysis, and present proportion of symptoms and estimate their 95% confidence intervals (CIs) using the exact method. When more than two studies provide information on a symptom, we will perform a meta-analysis using a random intercept logistic regression model. Heterogeneity between estimates will be assessed using the I ^2^ statistic.

Where data is available, we will explore key factors that affect prevalence estimates, e.g. hospitalisation, settings, location of the study, sex and follow-up timing using subgroup analysis and meta-regression analysis. We will identify these factors by discussing with our clinical experts and patient advocates and by reviewing the literature. The division for key factors used in subgroup analysis will depend on the availability of reported data across studies. We will align the division with the literature and expert opinions to form exploratory analysis and to help the interpretation. 

We will also conduct sensitivity analysis to examine the impact of high risk of bias studies and conventional statistical methods on the prevalence estimates, e.g. Freeman-Tukey Double arcsine transformation using inverse variance meta-analysis. All analysis and data presentation will be performed using meta and ggplot2 in R (version 4.0.5 or above) via RStudio (version 1.3.1093 or above).

We will work with patient advocates to present the data to facilitate transcription to lay audiences.

### Protocol registration

This protocol report is structured according to the Preferred Reporting Items for Systematic Reviews and Meta-Analyses Protocols (PRISMA-P) statement guidelines ^14^, was registered with PROSPERO ( CRD42020211131, 25 September 2020). The protocol will be updated as we progress with the living review as and if needed. CS is the guarantor for this study.

## Data availability

### Underlying data

No underlying data are associated with this article.

### Reporting guidelines

Figshare: PRISMA-P checklist for “What are the long-term symptoms and complications of COVID-19: a protocol for a living systematic review”. https://doi.org/10.25383/city.13187456.v1 ^15^.
