# Supplementary material for: What are the long-term symptoms and complications of COVID-19: a protocol for a living systematic review
Source: F1000Res. 2021 Aug 27;9:1455. Originally published 2020 Dec 14. [Version 2] doi: 10.12688/f1000research.27284.2 (PMC8459620; doi:10.12688/f1000research.27284.2)
Supplement: Peer review history

# Peer review: Reviewer response for version 2

**DOI:** 10.5256/f1000research.58402.r92984

**Published:** 2021-09-22

**Authors:** Madelon van Wely

**Affiliations:** 1 Centre for Reproductive Medicine, Amsterdam University Medical Centre, University of Amsterdam, Amsterdam, The Netherlands

In my view all queries were answered and adequately addressed, the protocol quality has improved and I am content with the present version. I wish the authors good luck with their review.

Is the study design appropriate for the research question?

Yes

Is the rationale for, and objectives of, the study clearly described?

Partly

Are sufficient details of the methods provided to allow replication by others?

No

Are the datasets clearly presented in a useable and accessible format?

Partly

Reviewer Expertise:

Epidemiology, meta-analyses, Obstetrics and Gynaecology

I confirm that I have read this submission and believe that I have an appropriate level of expertise to confirm that it is of an acceptable scientific standard.

# Peer review: Reviewer response for version 2

**DOI:** 10.5256/f1000research.58402.r92985

**Published:** 2021-08-31

**Authors:** Johannes Siegrist

**Affiliations:** 1 Senior Professorship on Work Stress Research, University of Duesseldorf, Duesseldorf, Germany

I have read the revision of the manuscript and approve the publication of this manuscript.

Is the study design appropriate for the research question?

Yes

Is the rationale for, and objectives of, the study clearly described?

Yes

Are sufficient details of the methods provided to allow replication by others?

Partly

Are the datasets clearly presented in a useable and accessible format?

Not applicable

Reviewer Expertise:

Social epidemiology systematic reviews

I confirm that I have read this submission and believe that I have an appropriate level of expertise to confirm that it is of an acceptable scientific standard.

# Peer review: Reviewer response for version 1

**DOI:** 10.5256/f1000research.30148.r80913

**Published:** 2021-03-22

**Authors:** Madelon van Wely

**Affiliations:** 1 Centre for Reproductive Medicine, Amsterdam University Medical Centre, University of Amsterdam, Amsterdam, The Netherlands

No doubt this is an important LSR that needs to be performed. I am in favor of getting this protocol published, however, at present many details are lacking. In my view in LSRs it is crucial to decide beforehand what the authors plan to do. Though it may seem annoying now, it will be helpful in the end. 

1. The outcomes. You performed a rapid review so you will know what main outcomes you are looking for. The outcomes are not stated in the Prospero protocol either.
2. It would also be advisable to have list of inclusion criteria and data you want to extract like risk factors and demographics. You could combine these with outcomes and present them in one table.
3. Data analysis: descriptive analysis sub grouped by age, sex, comorbidities? As the idea is to provide proportions from different studies I think you will be able to pool these, stratified by age etc. You could for instance summarise proportions (maybe use Freeman-Tukey transformation to stabilise variances in case of studies with zero events?), provide 95% CIs and predictive intervals to report on the precision of estimates. (meta-analysis of proportions)
4. About the subgrouping. How are you planning to do so? Are the age groups pre-specified or will they be dichotomised of subgrouped on basis of distribution of the data?
5. The duration is very important, how do you intend to present time? As grouped or continuous variable? When you have adequate data you could present the prevalence estimates over time.
6. Besides prevalence studies you might also include cohorts in which case you may want to assess the risk of bias using the Newcastle Ottawa Scale for comparative cohorts or another such checklist.
7. Search: I would also search specifically for the expected outcomes
8. I do advise to clarify when you consider a symptom to be persistent? You write: persistent mild, moderate or severe symptoms as defined by the article authors. I suppose post-discharge is T0.
9. How often do you at this moment intend to update and publish the results? 

Is the study design appropriate for the research question?

Yes

Is the rationale for, and objectives of, the study clearly described?

Partly

Are sufficient details of the methods provided to allow replication by others?

No

Are the datasets clearly presented in a useable and accessible format?

Partly

Reviewer Expertise:

Epidemiology, meta-analyses, Obstetrics and Gynaecology

I confirm that I have read this submission and believe that I have an appropriate level of expertise to state that I do not consider it to be of an acceptable scientific standard, for reasons outlined above.

# Response

**Published:** 2021-07-03

**Authors:** Charitini Stavropoulou

**Affiliations:** City, University of London, UK

Thank you for your thorough review, please find an outline of the response to the comments raised.

1. The outcomes. You performed a rapid review so you will know what main outcomes you are looking for. The outcomes are not stated in the Prospero protocol either. 

***Response:*** *Thanks for highlighting, we have updated the text in the methodology section to highlight that the primary outcome is to characterise the prevalence of symptoms and complications of long term Covid-19 in different populations. Secondary outcomes include diagnostics and risk factors for developing different sequelae. This is now stated on page 7 of the revised protocol.*

1. It would also be advisable to have list of inclusion criteria and data you want to extract like risk factors and demographics. You could combine these with outcomes and present them in one table. 

***Response*** ***:*** *We have provided further details in the methods, regarding the inclusion/exclusion criteria. We have also expanded on the data extraction section to show the clarify the data we will be extracting. Please see page 7 of the revised protocol.*

1. Data analysis: descriptive analysis sub grouped by age, sex, comorbidities? As the idea is to provide proportions from different studies I think you will be able to pool these, stratified by age etc. You could for instance summarise proportions (maybe use Freeman-Tukey transformation to stabilise variances in case of studies with zero events?), provide 95% CIs and predictive intervals to report on the precision of estimates. (meta-analysis of proportions) 

***Response*** *We have provided further information in our analysis section to clarify that the analysis will go beyond descriptive statistics and that we will perform meta-analysis wherever this is possible, i.e. when there are more than two studies providing information on a symptom. Please see pages 7 and 8 of the revised protocol. *

1. About the subgrouping. How are you planning to do so? Are the age groups pre-specified or will they be dichotomised of subgrouped on basis of distribution of the data?

***Response:*** *We plan to conduct subgroup analysis to explore the influence of key factors, e.g. age, on the estimates of prevalence. We will identify these key factors by discussing with our clinical experts, patient advocates and by reviewing the literature. We will try to align the division for subgroups with the literature to help inform the analysis and results. However, the methods will heavily depend on the availability and distribution of data. We have updated the methodology section accordingly.*

  

1. The duration is very important, how do you intend to present time? As grouped or continuous variable? When you have adequate data you could present the prevalence estimates over time. 

***Response:*** *We now clarify in the methods section that a subgroup analysis will be performed on follow up timing as indeed this is an important parameter as a group variable. *

1. Besides prevalence studies you might also include cohorts in which case you may want to assess the risk of bias using the Newcastle Ottawa Scale for comparative cohorts or another such checklist.

***Response:*** *We will include any study design including cohort studies with symptom prevalence. We have chosen to use the Hoy et al risk of bias assessment checklist, a validated tool for assessing risk of bias in prevalence studies, which does not prevent us from using it with cohort studies.*

1. Search: I would also search specifically for the expected outcomes

*Thank you for your suggestion. We have conducted pilot searches and noticed considerable diversity in the reported outcomes. The inclusion of key terms for the expected outcomes in search strings generated excessive hits and contributed to low precision, aka a low percentage of useful publications found in the search results. It also limits searches by “expected” outcomes and loses the opportunities finding new types of long covid symptoms and risk factors. Therefore, we used a search strategy using simple search strings focusing on describing long covid and complement the searches by backwards snowball searches in the references of relevant publications and consulting experts.   *

1. I do advise to clarify when you consider a symptom to be persistent? You write: persistent mild, moderate or severe symptoms as defined by the article authors. I suppose post-discharge is T0.

***Response.*** *Thanks for highlighting. We have provided further clarification in the inclusion criteria (page 5).  In light of the new definitions issued by the Office for National Statistics (ONS), we clarify we will be including studies assessing symptoms or outcomes at 12 or more weeks post Covid-19 onset.  *

1. How often do you at this moment intend to update and publish the results? 

***Response:*** *We will update the review every 6 months, as now clarified on page 5 of the methods section.*

# Peer review: Reviewer response for version 1

**DOI:** 10.5256/f1000research.30148.r78755

**Published:** 2021-02-12

**Authors:** Johannes Siegrist

**Affiliations:** 1 Senior Professorship on Work Stress Research, University of Duesseldorf, Duesseldorf, Germany

This protocol paper describes the aims and methods of conducting a living systematic review (LSR) on findings from studies following COVID-19 patients with long-terms symptoms. The LSR is planned to be updated at 2-months intervals up to 2 years. The paper offers information on search strategy with key terms, data management procedure, screening with critical appraisal, data extraction, and strategies of data analysis. Overall, the approach is in line with the PRISMA guidelines of performing systematic reviews and meta-analyses. Moreover, the protocol was registered in PROSPERO. Critical appraisal was proposed according to Hoy *et al.* 2012, and authors are expected to ensure that relevant criteria of risk of bias can be assessed by this tool.

 In summary, while the indexing of the paper is endorsed, several minor queries still need to be addressed, as detailed below.

**Search strategy:**

 Authors claim that their search will not be restricted by country and language. However, it is unrealistic to identify and analyse written materials in more than 3 or 4 main languages, and these languages (English plus…) should be explicitly mentioned in the study protocol.

**Data extraction:**

 I wonder why no data on treatment (therapy of COVID-19 symptoms) are included in the data extraction matrix. This information is crucial if aspects such as symptom severity and duration are being evaluated.

**Data analysis:**

 This section is not well elaborated. Basic descriptive statistics only are mentioned. However, systematized data from respective publications will probably allow additional, more informative ways of synthesizing the data.

Is the study design appropriate for the research question?

Yes

Is the rationale for, and objectives of, the study clearly described?

Yes

Are sufficient details of the methods provided to allow replication by others?

Partly

Are the datasets clearly presented in a useable and accessible format?

Not applicable

Reviewer Expertise:

Social epidemiology systematic reviews

I confirm that I have read this submission and believe that I have an appropriate level of expertise to confirm that it is of an acceptable scientific standard, however I have significant reservations, as outlined above.

# Response

**Published:** 2021-07-03

**Authors:** Charitini Stavropoulou

**Affiliations:** City, University of London, UK

*Thank you for your thorough review of our protocol, please find the response to the comments made outlined below.*

**Search strategy:**

 Authors claim that their search will not be restricted by country and language. However, it is unrealistic to identify and analyse written materials in more than 3 or 4 main languages, and these languages (English plus…) should be explicitly mentioned in the study protocol.

***Response:*** *The main search will be performed in English, articles identified in non-English languages will be translated using Google translate and assessed by a reviewer with good knowledge of the language. This is now stated on page 7 of the revised protocol.*

**Data extraction:**

 I wonder why no data on treatment (therapy of COVID-19 symptoms) are included in the data extraction matrix. This information is crucial if aspects such as symptom severity and duration are being evaluated.

***Response:*** *Thank you for the comment. This is part of the information included in the characteristics of population and is now explicitly mentioned in the protocol. Where data is available and synthesizable, we plan to assess the association between acute treatment and symptom severity and duration.*

**Data analysis:**

 This section is not well elaborated. Basic descriptive statistics only are mentioned. However, systematized data from respective publications will probably allow additional, more informative ways of synthesizing the data.

***Response:*** *Thank you for your comments. We have amended the data analysis section to clarify that the analysis will go beyond descriptive statistics and, when possible, we will perform a meta-analysis. Please see pages 7 and 8 of the revised protocol.*
